# State Laws to Regulate the Practice of Dentistry

**Published:** 1868-07

**Authors:** 


*State Laws to Regulate the Practice of Dentistry.*—Ohio and New York have now laws regulating the practice of dentistry within their limits. The Ohio law is everything which could be desired, and reflects credit upon its framers, while the New York law is sadly deficient and almost inert, as its framers will find after some little experience in its working. It is but just however to state that the committee of the Dentists of New York State claim to have "aimed at first for a good legal foundation to stand upon, and then to go on and more easily obtain such aditional legislation as they may need, with much less trouble than was had in the first law."

The Ohlo law which was passed by the Legislature, reads as follows:

"SECTION 1. *Be it enacted by the General Assembly of the State of Ohio*, That it shall be unlawful for any person to practice Dentistry in the State of Ohio for compensation, unless such person has received a Diploma from the faculty of a Dental College duly incorporated under the laws of this or any other State of the United States, or foreign country, or a certificate of qualification issued by the State Dental Society, or by a local society auxiliary thereto; provided, that nothing in this section shall apply to persons now engaged in the practice of Dentistry in this State, before the first day of January, 1873.

SEC. 2. Any person who shall practice Dentistry without having complied with the regulations of this act, shall be deemed guilty of a misdemeanor, and upon conviction thereof, shall be fined not less than fifty dollars no r more than two hundred dollars; provided, that nothing in this act shall be construed to prevent physicians and surgeons from extracting teeth.

SEC, 3. All prosecutions under this act shall be by indictment before the Court of Common Please in the County where the offense was committed, and all fines imposed and collected under the provisions of this act, shall be paid into the Treasury of the County where such conviction shall take place. for the use of the Common Schools within such County.

SEC. 4. This act shall take effect and be in force from and after its passage.''

We present as much of the New York law as we have have space for, and will interest our readers generally : The first seven sections relate to the places of meeting in the several judicial districts, the election of members and delegates, allowing each incorporated Dental College to elect two delegates.

SECTION 8. The State Dental Society, organized as aforesaid, at its first meeting shall appoint eight censors, one from each of the said district societies, who shall constitute a board of censors, and at the first meeting of said board the members shall be divided into four classes, to serve one, two, three and four years respectively, and said State Dental Society shall, at each annual meeting thereafter, appoint two censors, to serve each four years and until their successors shall be chosen, and fill all vacancies that may have occurred in the board by death, or otherwise. Each district society shall be entitled to one and only one member of said board of censors. Said board of censors shall meet at least once in each year, at such time and place as they shall designate, and being thus met, they, or a majority of them, shall carefully and impartially examine all persons who are entitled to examination under the provisions of this act, and who shall present themselves for that purpose, and report their opinion in writing to the president of said State Dental Society, and on the recommendation of said board it shall be the duty of the president, aforesaid, to issue a diploma to such person or persons, countersigned by the secretary, and bearing the seal of said society.

SEC. 9. All dentists in regular practice at the time of the passage of this act, and all persons who shall have received a diploma from any dental college in this State, and all students who shall have studied and practiced dental surgery with some accredited dentist or dentists for the term of four years, shall be entitled to an examination by said board of censors. Deduction from such term of four years shall be made in either of the following cases:

1. If the student, after the age of sixteen, shall have pursued any of the studies usual in the colleges of this State, the period, not exceeding one year, during which he shall have pursued such studies shall be deducted.

2. If the student, after the age of sixteen, shall have attended a complete course of lectures of any incorporated dental or medical college in this State or elsewhere, one year shall be deducted.

SEC. 10. Every person on receiving a diploma from the State Dental Society shall pay into the treasury thereof the sum of twenty dollars, and on receiving a certificate of qualification from the dental society of any district the sum of ten dollars into the treasury thereof.

SEC. 11. The dental societies of the respective districts, and the dental society of the State, may purchase and hold such real and personal estate as the purposes of their respective corporations may require. The district societies each not exceeding in value the sum of five thousand dollars, and the State Dental Society not exceeding twenty-five thousand dollars in value.

SEC. 12 The respective societies herein provided for may make all needful by-laws, rules and regulations, not inconsistent with any existing law, for the management of the affairs and property of said societies respectively, and providing for the admission and expulsion of members, provided that such by-laws, rules and regulations of the respective district societies shall not be repugnant to nor inconsistent with the by-laws, rules and regulations of the State Dental Society.

SEC. 13. All dentists who shall have been in regular practice in this State, at the time of the passage of this act, and all persons who shall have received a certificate of qualification from any district society, shall be eligible to membership in said district societies.

SEC. 14. The dental Society of the State of New York shall be entitled to all the privileges and immunities granted to the medical societies of this State.

SEC. 15. This act shall take effect immediately.

*The law was signed by the Governor April* 7, 1868.

It will be observed that in the Ohio law, graduates of incorporated Dental Colleges are not required to pass an examination before a board of censors, but in the New York law no difference is made in this respect between graduates and non-graduates.

It is simply ridiculous to suppose that every, or even any, board of censors will be composed of men qualified to examine those who have been regularly educated in their profession, and we do not hesitate to say that such candidates will, in the majority cases, be better prepared to examine the board, than the board the candidates.

It is very evident that some, at least, of the framers of this New York law were men who do not enjoy the privilege of being graduates of Dental Colleges and are envious of those who do, or such a clause as the one we refer to would not appear in it. We feel very certain that before this law has been in operation very long, the utter folly of such a course will become apparent to all who are not egotists. After a careful examination we are disposed to believe that this law has been manufactured for the purpose of granting diplomas to those who could in no other way obtain them.

